# 

**Published:** 1854-01

**Authors:** 


### INDEX TO VOLUME I.

Absorption, Chloride Sodium, - 149
Acid Sulph. in Diarrhea, - - 316
Air Passages, Foreign Bodies in, 242
Alcohol, Carpenter on, - - - 303
Articular Rheumatism, - - 245
Asylum Lunatic, N. Y., - - - 308
Arsenic, Poisoning by, - - - 320
Antidote, Magnesia, - - - - 147
American Med. Association, 78—437
American Medical Literature, - 465
Anatomy, Richardson's, - 80—195
Antisell's Address, - - - - - - 379
Ascites Mistaken, Death, - - - 190
Asylums, Insane, - - - - - - 383
Atropia, Poisoning, - - - - - 163
Ayres, Diphtheritis, - - - - - 276
Blood, Morbid Matter in, - - - 318
Breast Tumor, - - - - - - - 243
Breckinridge's R. J. Address, - 304
Belladonna in Pneumonia, - - 402
Bullock's Blood, - - - - - - 404
Calculus Uniary, - - - 264—273
Chloride Sodium, Absorption, - 149
Cholera, - - 139—159—160—383
Chlorate Potash in Croup, - - 244
Chloroform, - 162—349—399—479
Cooke, Dr. J. E., Obituary of, - 82
Chenoweth on Pleurodynia, - - 352
Cinnamon, Menorrhagia, - - - 153
Croup, - - - - - 235—244—397
Crania, Flat Head Indians, - - 233
Coffee, Physiological Effects, - - 309
Consumption, - - - - - - - 435
Cataract, - - - - - - - - - 478
Diabetes Melitus, - - - - - 479
Diarrhea, Sulphuric Acid, - - 316
Diphtheritis - - - - - - - - 276
Dowler on Yellow Fever, - - - 279
Dysmenorrhœa, Biborate Soda, - 164
Dyspepsia, - - - - - - - - 238
Dysentery, Recurrence, - - - 317
Emphysema, Flint's Cases, - - 325
Encephaloid, Mammary, - - - 173
Eyelids, Burn by Lime, - - - 181
Felt Splints, - - - - - - - - 162
Fœtus, Secondary, - - - - - 119
Fever, Stokes on, - - - - - 225
Flint on Pleurisy, - - - - - 23
Flint on Pericarditis, - - - - 86
Flint on Rheumatism, - - - - 245
Flint on Emphysema, - - - - 325
Flint, Lettor from Paris, - - - 405
Fracture of Humerus, - - - - 54
Fracture Cervical Vertebra, - - 158
Fenner on Yellow Fever, - - - 279
Fevers, - - - - - - - - - - 479
Gonorrhea, - - - - - - - - 480
Gout, - - - - - - - - - - 480
Gross, Univer. Clinic., 48—103—264
Headache, - - - - - - - - - 469
Heart, Hypertrophy, - - - - 421
Hernia, Scrotal, - - - - - - 165
Hernia, Femoral, - - - - - 185
Humerus, Fracture of, - - - - 54
Hydrocele, Operation with Seton, 49
Homœopathy, - - 143—211—385
Hospital for Insane, Tenn. - - 223
Horner, Prof., Notice of, - - - 141
Hysterical Monomania, - - - 229
Hypertrophy, Heart, - - - - 421
Inversion of Uterus, - - - - 95
Indian Skulls, - - - - - - - 233
Infusoria in Milk, - - - - - 323
Iodide Iron, Extemporary, - - 313
Iron Perchloride - - - - - - 481
Jesse, Secondary Fœtus, - - - 119
Kentucky Medical Society, 224—298
Lancet, London, - - - - - - 306
Lead Poisoning, - - - - - 480
Lithotomy, - - - - - 264—273
Lithotrity. forty-eight Time, - 318
Lime Phosphate, - - - - - - 482
Lunatic Asylum, N. Y. - - - 308
Lungs, Emphysema of, - - - 325
Malpractice, Law in, - - - - 389
Mamma, Encephaloid of, - - - 173
Mamma, Schirvus, - - - - - - 117
Medicine, its Mission, - - - - 5
Medical Association, - - - - 78
Medicine, Eclectic, - - - - - 296

Menorrhagia, - - - - - - - 480
Menorrhagia, Cinnamon, - - - 153
Midwifery, Chloroform, - - - 399
Mortality, St. Louis, - - - - 314
Milk, Infusoria in, - - - - - 323
Mitchell, Case Hypertrophy, - 421
Medical Literature, - - - - 465
Meigs on Woman, - - - - - 433
Neuralgia, Traumatic, - - - 59—480
Ointment Chloroform, - - - - 349
Onychia Maligna, - - - - - 70
Ophthalmia, Strumous - - - 103
Palmer's Introductory Lecture, - 5
Pericarditis with delirium, - - 86
Pleurisy, Flint on, - - - - - 23
Pleurodynia, - - - - - - - 352
Physiological Chemistry, - - - 123
Physician's Life, Pleasures of, - 145
Phthisis, Signs, Incipient, - - 155
Poisoning, Arsenic, - - - - - 320
Poisoning, Atropia, - - - - 163
Pneumonia, Belladonna, - - - 402
Pills, Large Doses Children, - - 403
Perchloride Iron, - - - - - - 481
Pityriasis Capitis, - - - - - 482
Phthisis, - - - - - - - - - 482
Pneumonia, - - - - - - - 482
Re-vaccination, Necessity of - - 147
Richardson's Anatomy, - - - 80
Rheumatism, Veratria, - - - 162
Rheumatism, Flint's Cases, - - 245
Rogers, Inversion Uterus, - - 95
Ronald on Chloroform Ointment, 349
Rheumatism, Fuller on, - - - 428
Sciatica, - - - - - - - - - 483
Scrotal Hernia, - - - - - - - 165
Silliman on Phys. Chem. - - 123
Simpson on Homœopathy, - - 211
Small Pox, - - - - - - 147—387
Spermatorrhea, - - - - - - - 483
Splints, Felt, Hamilton, - - - 162
Strabismus, - - - - - - - 483
Spleen, Tumor of, - - - - - - 483
Sulphuric Acid, Diarrhea, - - 316
Surgeon, Heroic Conduct of - - 324
Syphilis, - - - - - - - - - 484
Tapeworm, - - - - - - - - 484
Tracheotomy, - - - - - - 397
Traumatic Neuralgia, - - - - 59
Tumor, Fatty, - - - - - - - 62
Tumor, Hydatid on Arm, - - 116
Ulcers, - - - - - - - - - - 484
University Clinic, - - - - - 48
Uterus, Inversion of - - - - 95
University, Louisville, - - - 467
Vertebra, Cervical, Fracture, - 158
Vomiting, - - - - - - - - - 484
Weather, - - - - - - - - - 306
Williams' Letters from Paris, - 181
Woman, Meigs on, - - - - - - 433
Yellow Eever, - - - - 279—351

### The Western Journal

OF

### MEDICINE AND SURGERY.

### NEW SERIES.

The Old Series of this Periodical, consisting of Twenty-eight volumes, was brought to an abrupt termination, by the destruction of the "Louisville Journal" office, by fire, in November, 1853. Arrangements have been made with Mr. J. F. Brennan, Publisher, for its continuation, under more favorable auspices, and in a much improved form. It has been considered prudent to reduce the price of the work to subscribers from $5 to $3 per annum, payment to be invariably made in advance, and each subscription to commence with the present number,—a supply of which has been printed sufficient to satisfy all presumable demand. The Editor guarantees to procure of his colleagues, in the University of Louisville, articles for each number, which, with his own productions and selections from the leading Medical publications of Europe and America, will, he flatters himself, afford the subscribers and readers of the Western Journal, a monthly periodical readable and instructive, and well worth the price at which the publisher supplies it.

The arrangement made with the new publisher of this work does not demand of him responsibility for the failure, in any way, of the old issue. This is distinctly understood. Such responsibility will be met by the late publisher, or his assigns; and communications upon that subject should be so directed, to receive attention.

The present issue of this work will consist of Eighty pages a month. With the closing or December number, a title-page and copious Index will be supplied; and the publisher will be prepared to bind the work in a handsome uniform manner, in embossed muslin, at a small cost.

☞ All communications, containing subscription or any other business matter connected with the publishing department, should be addressed to

J. F. BRENNAN, Box 126, Louisville, Ky.

and all pertaining to the editorial department, to the Editor, L. P. YANDELL, M. D., Louisville, Ky.

☞ Subscription, Three Dollars per annum. No name entered until the money is received.

